# Automated quantification of Epstein-Barr virus in whole blood for post-transplant lymphoproliferative disorders monitoring

**DOI:** 10.1186/s12985-020-1285-7

**Published:** 2020-02-03

**Authors:** Maud Salmona, Karl Stefic, Nadia Mahjoub, Flore Sicre de Fontbrune, Sarah Maylin, François Simon, Catherine Scieux, Gérard Socié, Marie-Christine Mazeron, Jérôme LeGoff

**Affiliations:** 1 Université de Paris Diderot, INSERM U976, Paris, France; 2 Laboratoire de Microbiologie, Hôpital Saint-Louis, APHP, Paris, France; 3 Université de Tours, INSERM U1259, Tours, France; 4 Hematology and Transplantation Unit, Hôpital Saint-Louis, APHP, Paris, France

**Keywords:** EBV, Quantitative PCR, Hematopoietic stem cell transplant, Monitoring, Rituximab

## Abstract

**Background:**

Standardized and sensitive assays for Epstein Barr Virus (EBV) are needed to define universal cutoff for treatment initiation in allogeneic hematopoietic stem cells transplant recipients. In a context of accreditation and the availability of EBV international standard, we evaluated the Abbott RealTime EBV (RT) assay for EBV quantification in whole blood.

**Methods:**

The RT assay was compared on 282 prospective clinical samples with the Artus EBV PCR Kit V1 assay (V1) and we analyzed the kinetics of EBV load in 11 patients receiving rituximab treatment.

**Results:**

The estimated limit of detection was 88 IU/mL. The assay was linear (r^2^ = 0.9974) in the range of all samples tested (100 to 1,000,000 IU/mL). Intra-assay coefficients of variation (CV) ranged between 0.35 and 1.35%, and inter-assay CV between 3.40 and 4.5%. On samples above the limit of quantification, the two assays were strongly correlated. EBV RT values were on average 0.30 log_10_ IU/mL lower than those measured with the V1 assay. In patients treated with rituximab, the RT assay remained positive in 5 patients at the time it dropped below undetectable levels with the V1 assay.

**Conclusions:**

In conclusion, the RT assay is a reliable assay for EBV load in whole blood. Its sensitivity will enable to estimate the kinetics of EBV load and the impact of treatments to control EBV reactivations.

## Background

Epstein Barr Virus (EBV) is a member of the Herpesviridae family. It is estimated that this ubiquitous virus infects more than 90% of the world’s population [1]. After infection, EBV establishes a life-long persistent and latent infection in B lymphocytes. EBV is the causative agent of infectious mononucleosis and is associated with different hematological and epithelial malignancies [2, 3]. In immunocompromised patients, especially allogeneic hematopoietic stem cell (HSCT) or solid-organ transplant recipients, EBV is associated with post-transplant lymphoproliferative disorders (PTLD) [4, 5].

Quantification of EBV DNA load in blood is used to identify patients at risk of PTLD [6–8] and EBV DNA levels are considered to start preemptive therapies, including lowering immunosuppressants and administering anti-CD20 monoclonal antibodies, such as rituximab. As EBV DNA load in blood is taken into account for preemptive strategies, accurate and reliable quantification is necessary for the management of patients after transplant [9–13]. However, no universal quantitative cutoff of EBV DNA load to start preemptive treatment has been defined yet. Furthermore, only few studies describe the dynamics of EBV DNA load after rituximab injection [14] .

Results of measurements of EBV DNA loads performed with commercially available assays might differ significantly, particularly according to the extraction method used, which is a source of variability with whole blood (WB) [15]. Several studies have reported significant inter-laboratory variation in EBV DNA quantification both in whole blood and plasma samples [16–18].

The determination of a consensual quantitative threshold requires standardization between techniques. In this context, there is a trend for the use of tests approved by regulation authorities, in particular IVD/CE labeled in Europe or FDA approved commercial assays in the USA, and automated techniques [15–21].

Furthermore, since 2010, French national regulations of clinical laboratories require that all the biological assays must be accredited according to the International Standard ISO 15189 [22]. In order to harmonize results and overcome the variability between laboratories, the World Health Organization (WHO) Expert Committee on Biological Standardization established recently the first international standard (IS) for EBV for nucleic acid amplification techniques [23]. Reporting EBV DNA quantification in international unit (IU) is now recommended and industrials develop their assays according to this international standard.

In this study, we performed an evaluation of the IVD/CE labeled Real-time EBV assay Abbott which includes a fully automated extraction and amplification of EBV DNA from whole blood on the m2000 Real-time systems according to the ISO 15189 recommendations. Clinical samples were tested and the results compared to those obtained with the Artus EBV kit V1 on the m2000 RealTime system [20] that Abbott commercialized before the development of their own assay. We also report the kinetics of EBV load in HSCT patients receiving rituximab therapy.

## Materials and methods

### Standard and clinical samples

#### Ethical considerations

The study was carried out in accordance with the Declaration of Helsinki. This study was a non-interventional study with no addition sampling to usual procedures. Biological material and clinical data were obtained only for standard viral diagnostic following physicians’ prescriptions (no specific sampling, no modification of the sampling protocol). Data analyses were carried out using an anonymized database. According to the French Health Public Law (CSP Art L 1121–1.1), such protocol was exempted from informed consent application.

#### Clinical samples

Leftover EDTA K2 tube (BD Vacutainer®) samples sent to the Virology unit of Saint Louis Hospital for EBV monitoring were used in this study. A total of 282 whole blood (WB) specimens received in the laboratory for EBV load quantification were collected from 196 patients including 95 hematopoietic stem cell transplant recipients, 22 kidney transplant recipients, 29 patients with immunological or heamatological diseases, 27 patients from general medicine, 10 HIV infected patients, 9 patients from intensive care unit and 4 patients hospitalized in infectious disease department. The clinical samples were selected prospectively and retrospectively from aliquots frozen at − 80 °C. All clinical consecutive whole blood samples received for EBV quantification within 7 days (124) were tested with the two assays. In order to obtain sufficient positive samples to enable a correlation analysis of EBV loads between the two assays, additional positive whole blood samples were selected retrospectively within 8 months. The samples were tested in separate runs both with the Abbott RealTime EBV assay (RT assay) and EBV PCR Kit V1 assay (V1 assay). Furthermore, 75 additional frozen samples at − 80 °C of 11 HSCT recipients were tested in order to analyze EBV DNA kinetics after rituximab injection in the two quantitative real time PCR assays.

#### AcroMetrix™ EBV controls high and low

Thermo Scientific™ AcroMetrix™ EBV low positive-control (catalog number 961230, lot 513,101) and high-positive-control (catalog number 961231, lot 417,503) were used for analytical evaluation of RT assay. The manufacturer’s instructions mentioned that expected results when using the AcroMetrix™ EBV Low and High controls must be established by the end user for their particular EBV DNA assay.

#### QCMD

The Quality Control for Molecular Diagnosis (QCMD) 2016 EBV WB challenge 1 and 2 and 2017 EBV WB challenge 1 and 2 proficiency panels were composed of 5 frozen WB samples. Expected values correspond to mean consensus (log_10_ IU/mL) calculated from data returned by participants from different laboratories after removing outliers.

#### WHO international standard

The first WHO international standard for EBV for nucleic acid amplification techniques (National Institute for Biological Standards and Control NIBSC code 09/260; Potters Bar, Hertfordshire, Great Britain) is a lyophilized whole virus preparation of the EBV B95–8 strain (type 1). The material has been assigned a concentration of 5 × 10^6^ IU/mL when reconstituted in 1 mL of nuclease-free water.

### Quantitative real time PCR assays

The quantification of EBV in WB was carried out on the Abbott m2000 platform for the two assays. This platform includes the m2000 sp. instrument for automated extraction of DNA and the m2000 rt. instrument for real-time PCR of series of 48 samples.

#### Abbott RealTime EBV assay (RT assay)

The amplification target is a highly conserved region of the BLLF1 gene which encodes the gp350/220 envelope glycoprotein. The Abbott RealTime EBV assay (Abbott Molecular Inc., Des Plaines, IL, USA) uses three reagent kits, the amplification reagent kit, the calibrator kit for the standard curve and the control kit for external control. An internal control is also supplied to check the overall internal process, including DNA extraction and possible PCR inhibition. Extraction of DNA was done on the m2000sp system. DNA extraction was performed from 300 μL of WB and eluted in 250 μL. Extraction was followed by automated addition of 25 μL of master mix and 35 μL of purified DNA into the PCR plate. In each run, one negative control and two positive controls (Low and High) were included. Two calibrators (A and B) were used to determine the standard curve. The results are expressed in IU/mL. Manufacturer lower limit of quantification (LLQ) is reported as 150 IU/mL and limit of detection (LOD) as 115 IU/mL.

#### Artus EBV PCR kit V1 assay (V1 assay)

EBV DNA quantification with the Artus EBV PCR Kit V1 assay (Qiagen, MD, USA, previously commercialized by Abbott Molecular) was also performed on the m2000 platform in batches of 48 tests. The PCR amplification reagent targets a conserved region within the gene coding for Epstein Barr virus Nuclear Antigen (EBNA1). The EBV PCR Kit V1 includes an internal control to check the overall process including DNA extraction and possible PCR inhibition. Automated DNA extraction and PCR reaction set up were performed on the Abbott *m*2000*sp* instrument. Briefly, DNA was purified from 300 μL of WB and eluted in 250 μL. The EBV quantification was performed with 20 μL of purified DNA. Sealed PCR plates were loaded on the Abbott *m*2000*rt* instrument for real-time PCR. Four calibrators (QS1, QS2, QS3 and QS4) were used to establish a calibration curve. Every run included one low calibrator (QS3). The results were expressed in copies/mL. For comparison with the RT assay, conversion factor previously calculated [20] was used to obtain IU/mL. The LLQ of the assay was 1000 copies/mL corresponding to 310 IU/mL.

### Quantitative real time PCR assays interpretation

For both assays, the results were classified as follows: target not detected, target detected but not quantifiable (< LLQ) and target detected and quantifiable (>LLQ and in the range of linearity).

### Analytical performances of the Abbott RealTime EBV assay

All dilutions were performed in EBV negative whole blood.

#### Limit of detection

The LOD was estimated by using serial dilutions of the WHO international standard at expected value of 500, 100 and 20 IU/mL. Each dilution was tested 10 times. The LOD is defined as the EBV DNA concentration detected with a probability of 95% or more.

#### Assay linearity

The assay linearity was verified with dilutions of a highly EBV DNA positive sample in EBV negative WB at expected value of 1,000,000, 100,000, 10,000, 1000 and 100 IU/mL. Each dilution was quantified with the RT assay 3 times and the mean EBV concentration of each sample was calculated.

#### Repeatability

The repeatability was determined with the AcroMetrix™ EBV Plasma Control High (4.76 log_10_ IU/mL) and two EBV DNA positive WB clinical samples (7.20 log_10_ IU/mL - “Blood High”- and 4.09 log_10_ IU/mL - “Blood Low” quantified with Abbott RealTime EBV assay). Ten replicates of each sample were tested in the same run. For each sample, intra assay coefficient of variation (CV) was estimated.

#### Reproducibility

The reproducibility was determined with the AcroMetrix™ EBV Plasma Control High and Low. Sixteen replicates of AcroMetrix High and Low were tested on a period of 16 days by four different operators. For each sample, inter assay CV was estimated.

#### Cross-contamination

A panel of 30 samples consisting of alternate phosphate buffered saline as negative samples and High-load EBV DNA WB (mean = 4.29 log_10_ IU/mL) were assayed on the three m2000 platforms.

### Statistical analysis

Concordance on qualitative results between the RT assay and the V1 assay was established by Cohen’s kappa statistic. The evaluation of quantitative correlation between the two assays included results positive in both and was estimated by using linear regression analysis and Bland-Altman plots. Statistical analysis was performed using GraphPad Prism6 software [24]. Differences were considered statistically significant at *p*-values below 0.05.

## Results

### Analytical performances of the Abbott RealTime EBV assay

The LOD was estimated by using 10 replicates for the three concentrations (500, 100 and 20 IU/mL) as shown in Table 1. The RT assay detected all replicates at 100 IU/mL. Probit analysis of the data predicted a LOD at 88 IU/mL. The assay was linear (r^2^ = 0.9974) in the range of all samples tested (1,000,000 to 100 IU/mL). Intra assay CV, determined on 10 replicates, were 1.35, 0.35 and 1.33% at the mean value of 4.80 log_10_ IU/mL (CI 95 4.75–4.84, AcroMetrix high), 7.30 log_10_ IU/mL (CI 95 7.28–7.32, Blood high) and 4.20 log_10_ IU/mL (CI95 4.159–4.239, Blood low). Inter assay CV, determined on 16 replicates were 4.5% at the mean value of 3.61 log_10_ IU/mL (CI 95 3.52–3.69) and 3.40% at the mean value of 4.98 log_10_ IU/mL (CI 95 4.89–5.07). Cross-contamination assays with a panel of 30 negative and highly positive alternate samples did not show any positive result in negative sample on the three m2000 platforms.

**Table 1:** Lower limit of detection (LOD) of the Abbott RealTime EBV assay for whole blood

Expected value (IU/mL)	No. of replicates	Mean value (IU/mL)	Detection rate (%)
500	10	1239	100
100	10	328	100
20	10	73	60

The LOD was estimated by using serial dilutions of WHO international standard in whole blood

### Results from the QCMD EBV proficiency panels

For quantitative analysis, our results were compared to the consensus mean and standard deviation calculated from all the data returned by the participants to the QCMD EBV proficiency panels. Differences between measured and expected values ranged from 0.254 (16C1–05) to − 0.443 log_10_ IU/mL (16C1–02) (median = 0.051 log_10_ IU/mL) with a correlation coefficient calculated on positive values of 0.8594 (*p* = 0.0002). EBV DNA was not detected in the negative QCMD EBV samples (16C1–03 and 16C2–03) and in the educational QCMD samples 16C1–04, 16C2–04 and 17C1–05 (respectively 2.389, 2.430 and 2.597 log_10_ IU/mL). EBV DNA was below the limit of quantification in the educational QCMD 17C1–02(2.457 log_10_ IU/mL) (Table 2).

**Table 2:** Quantification of EBV DNA with Abbott RealTime EBV assay in QCMD 2016 EBV samples

		Sample	QCMD results			RT EBV assay results	
			EBV load (log_10_ copies/mL)	n	Range	EBV load (log_10_ copies/mL)	Delta log_10_ (RT-QCMD)
2016 panel 1	C	16C1–01	3.673	52/52	2.937–4.772	3.819	0.146
	C	16C1–02	3.200	48/52	1.398–4.094	2.757	−0.443
	C	16C1–03	ND	N/A	N/A	ND	ND
	E	16C1–04	2.389	37/52	1.000–3.732	ND	ND
	C	16C1–05	3.673	52/52	3.247–4.580	3.927	0.254
2016 panel 2	C	16C2–01	4.144	53/53	3.511–4.710	4.096	−0.048
	C	16C2–02	2.949	49/53	2.068–3.781	3.074	0.125
	C	16C2–03	ND	N/A	N/A	ND	ND
	E	16C2–04	2.430	31/53	1.079–3.534	ND	ND
	C	16C2–05	3.684	53/53	3.117–4.302	3.539	−0.145
2017 panel 1	C	17C1–01	4.112	46/47	3.002–5.105	3.941	−0.171
	E	17C1–02	2.457	30/47	1.613–3.182	Pos NQ	ND
	C	17C1–03	4.103	46/47	3.548–5.240	3.994	−0.109
	C	17C1–04	3.573	44/47	2.444–4.454	3.435	−0.138
	E	17C1–05	2.597	27/47	1.519–3.872	ND	ND
2017 panel 2	C	17C2–01	4.220	50/50	3.609–4.837	4.179	−0.041
	C	17C2–02	3.661	50/50	3.029–4.754	3.497	−0.164
	–	17C2–03	Excluded			Excluded	ND
	C	17C2–04	3.660	49/50	3.208–4.089	3.609	−0.051
	C	17C2–05	3.009	47/50	2.107–3.877	3.079	0.07

Consensus log_10_ virus concentrations were calculated as the mean values reported from positives datasets (n) submitted by clinical laboratories

ND, EBV DNA was not detected

Pos NQ, EBV DNA was detected but not quantified

C core, E educational

Excluded, panel member was excluded of the QCMD report

delta log_10_, log_10_ copies/ml difference between the RT assay and QCMD consensus

### Comparison of the Abbott RealTime EBV and EBV PCR kit V1

A total of 282 WB clinical samples was analyzed using the two assays (Table 3). By using RT assay, DNA EBV was detected in 196 samples of which 176 were quantified above the LLQ value. With the V1 assay, EBV DNA was detected in 199 samples of which 173 were quantified above the LLQ value. One hundred eighty-six and 161 samples were respectively detected positive and quantified with both assays. The two assays showed a good agreement of 91.84% with a Kappa index of 0.81.

**Table 3:** Comparison Of V1 And Rt Ebv Assays On 282 Whole Blood Samples

		RT EBV assay			
		Quantified	Detected	Not Detected	Total
V1 assay	Quantified	161	8	4	173
	Detected	12	5	9	26
	Not Detected	3	7	73	83
	Total	176	20	86	282

Quantified: EBV DNA detected and above the limit of quantification of the assay

Detected: EBV DNA detected and below the limit of quantification of the assay

Results between the two techniques were discordant for 23 samples (8%). Discrepancies were observed mainly for samples with a low viral load. Thirteen samples were detected only with V1 assay (DNA load below the LLQ for 9 samples and at 2.59, 2.61, 2.64 and 2.66 log_10_ IU/mL for the four other samples) and 10 samples were detected only with RT assay (DNA load below the LLQ for 7 samples and at 2.18, 2.19 and 2.51 log_10_ IU/mL for the three other samples).

**Fig. 1:**
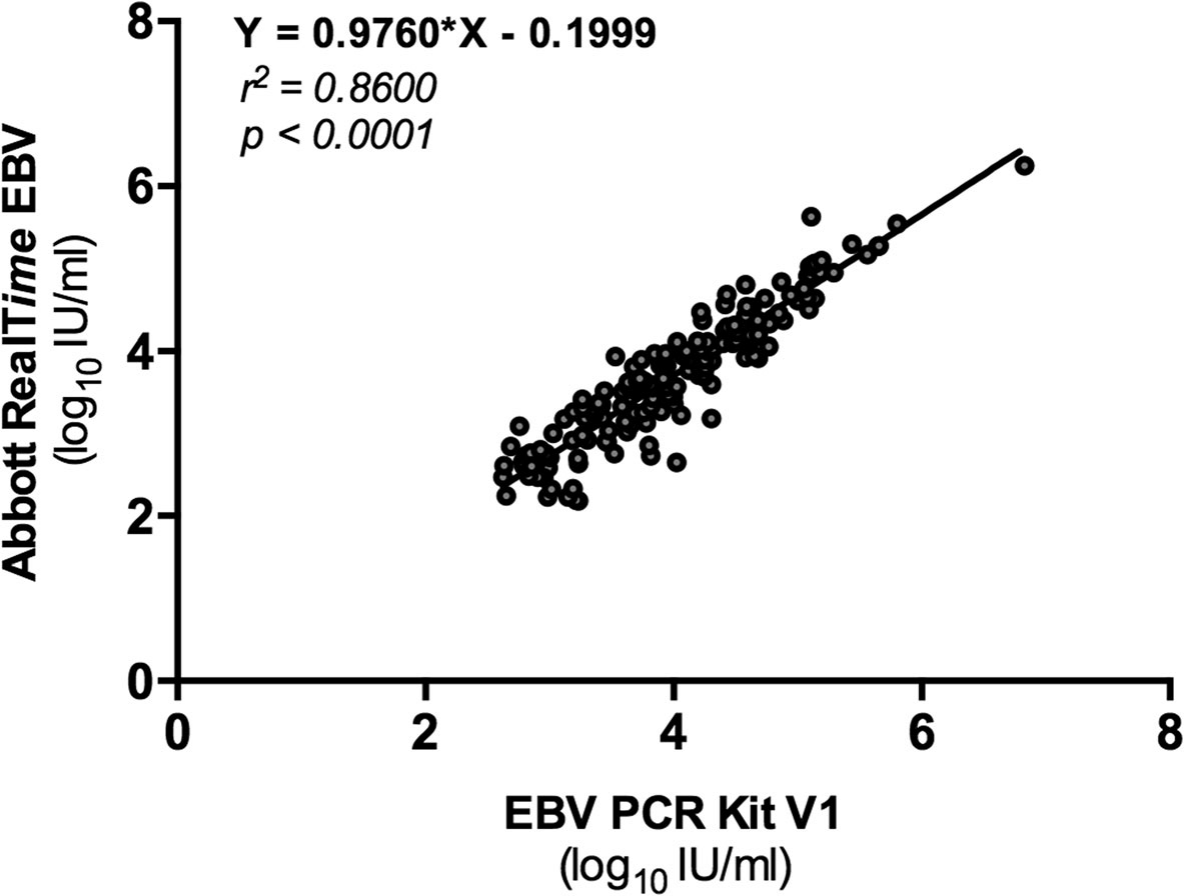
Correlation plot of EBV DNA load values measured by the two assays. Correlation plot of EBV DNA load values measured by EBV PCR kit v1 and the Abbott RealTime EBV assays for samples quantified with the two assays (n = 161). EBV DNA loads are expressed in log_10_ IU/mL. For V1 assay, log_10_ IU/mL were calculated from log copies/mL using previously calculated conversion factor

The analysis of the 161 samples quantified by both assays showed a r^2^ of 0.8600 between RT and V1 assay (*p* < 0.0001) (Fig. 1). Viral load values measured with the RT assay were on average 0.30 log_10_IU/mL (IC95 0.25–0.34) lower than those measured with the V1 assay (*p* < 0.0001).

**Fig. 2:**
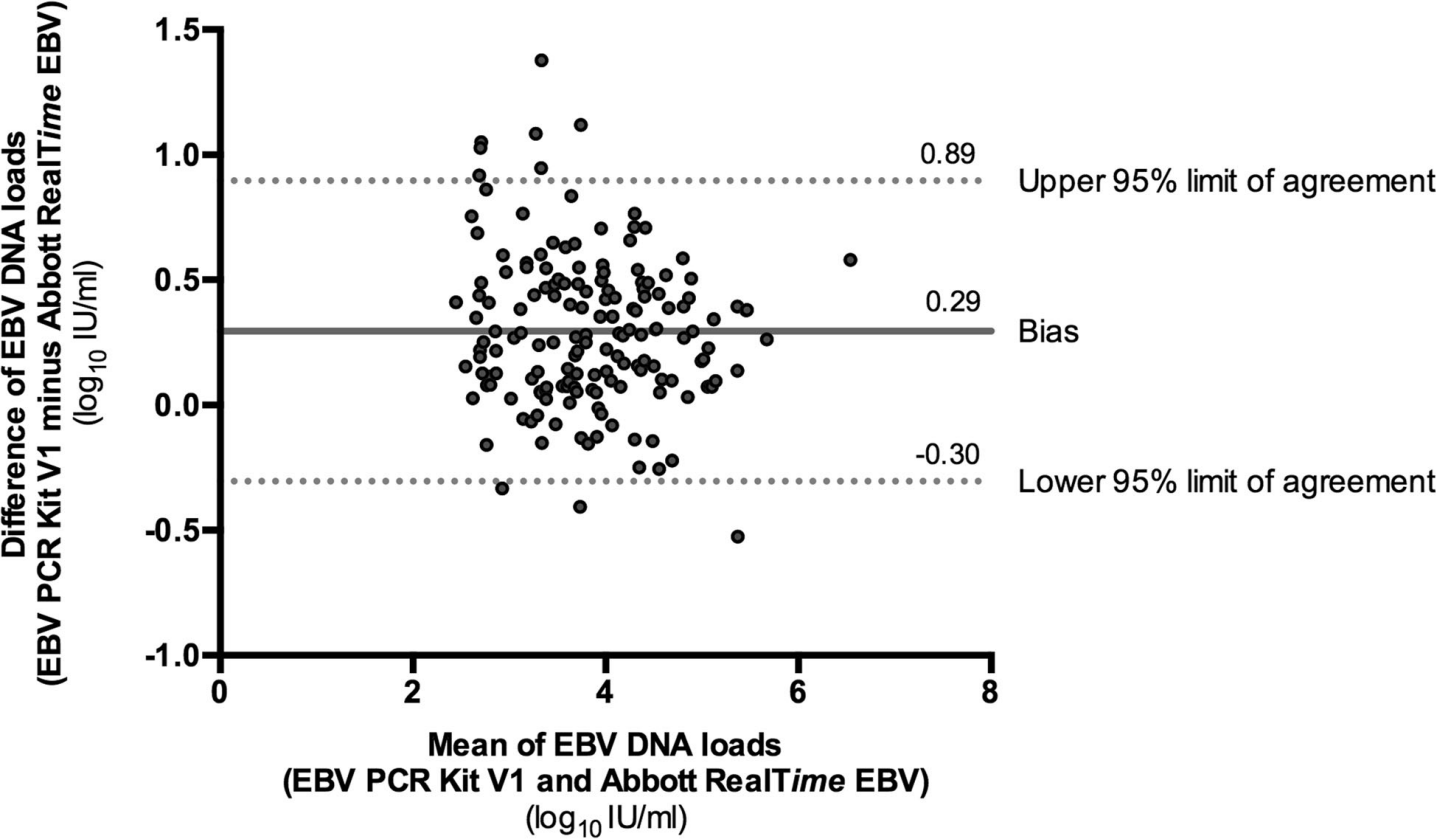
Bland-Altman plot of the two assays. Bland-Altman plot of the EBV PCR kit v1 and the Abbott RealTime EBV assays for samples quantified with the two assays (*n* = 161). EBV DNA loads are expressed in log_10_ IU/mL

Bland-Altman analysis was used to determine the limit of agreement between the two assays. The mean bias was 0.29 and 95% limits of agreement ranging from − 0.30 to 0.89. Ten samples were outside the 95% limits of agreement (Fig. 2). Among the 161 samples quantified by both assays, only one sample differed more than 0.5 log_10_ IU/ml between the two assays (5.11 log_10_ IU/mL with V1 assay and 5.63 log_10_ IU/mL with RT assay).

The internal controls included in both test systems were detected within the expected range in all samples.

### Kinetics of EBV DNA in WB in 11 HSCT after rituximab injection measured with both assays

**Fig. 3:**
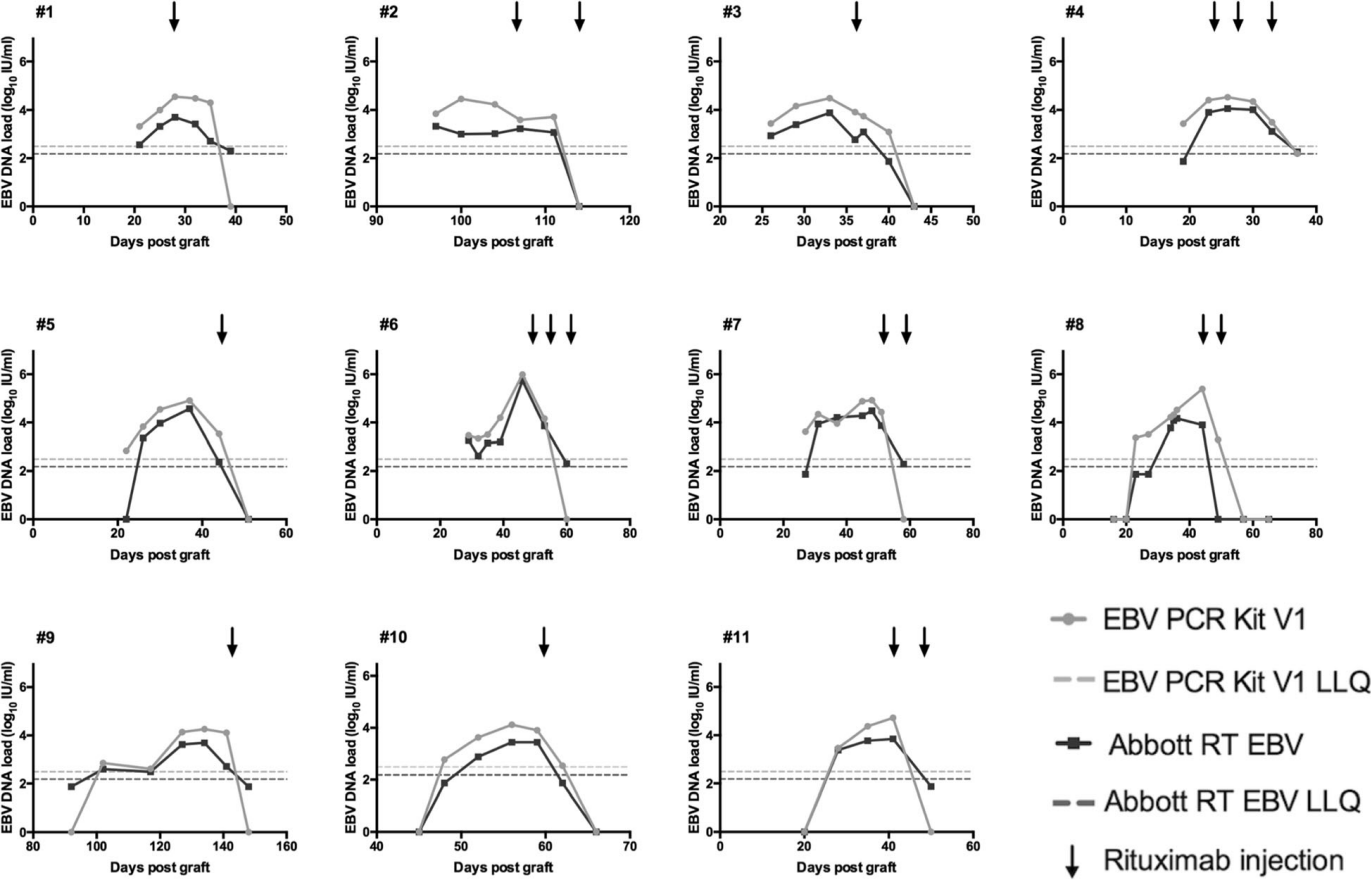
Kinetics of EBV DNA before and after rituximab injection measured with the two assays. Kinetics of EBV DNA was measured in 11 hematopoietic stem cell transplant recipients (#1 to #11) with the EBV PCR kit V1 and the Abbott RealTime EBV assay. EBV DNA loads are expressed in log_10_ IU/mL. Black arrows indicate time of rituximab injection. Detectable but non quantifiable samples are represented as the half of the LLQ of the technique. Non detectable samples are represented as zero on the graph

In order to further analyze the correlation between the two assays we compared the viral load kinetics for 11 HSCT recipients who had at least three successively positive samples and received rituximab. Our local practice recommended starting rituximab when EBV load in blood rose above 3.49 log_10_ IU/mL log_10_ with V1 assay after consideration of patient’s risk factors. Number of doses varied according the kinetics of EBV loads and biological and clinical parameters. As shown in Fig. 3, the profiles of the two assays were very similar for every patient and variations were always in the same direction. With the use of RT assay, EBV load was considered still positive in 5 patients at the time it dropped below undetectable levels with V1 assay. This may help to better estimate the kinetics of EBV load and the impact of treatments to control EBV reactivations. The median number of doses of rituximab was two. All patients responded to rituximab with a median EBV DNA load decrease after the first dose of 0.42 log_10_ IU/mL/day (range: 0.69 to − 0.06 log_10_ IU/mL/day; EBV load values with RT assay). In nine patients (#2, #3, #5–11), a significant decrease of EBV load (> 0.5 log; EBV load values with RT assay) was observed after the first dose while it remained stable in two patients (#1, #4). After a median follow-up of 517 days (range: 82 to 1468 days) none of them had developed a PTLD.

## Discussion

Sensitive and reproducible EBV DNA quantification in blood is critical for preemptive treatment initiation and monitoring. The medical community involved in the management of HSCT patients needs consensual threshold values to start treatments. To achieve this goal, the first step is the creation of an international standard for the quantification of EBV DNA. We have tested an automated assay for the quantification of EBV in whole blood using the WHO EBV international standard. In addition to the international standard we also tested QCMD panels to better estimate the accuracy of the assay. We then compared results from this assay with results from a former real-time quantitative PCR, a series of clinical samples and the prospective follow-up of EBV load in patients who received a therapy with rituximab for EBV reactivation.

The LOD estimated in our study (88 IU/mL) was close to that defined by the manufacturer (115.2 IU/mL, 95%CI 97.6–150.5 IU/mL) who also used WHO EBV international standard for LOD determination. Previous evaluation of this assay by Lee et al. estimated a lower LOD (48.9 IU/mL) that may be explained by the use of different analytical panels (Qnostic) [25]. The assay was linear in the large range of values (10^2^ to 10^6^ IU/mL) that fits with values usually observed in blood samples. The assay was highly reproducible with intra CV below 2% and inter CV below 5% which is similar to values previously reported [25]. In addition, the assay enables a complete traceability from sample registration in the laboratory informatics system (LIS) to results transmission from the molecular platform to the LIS. Altogether our results enabled to fulfill criteria to approve RT assay in accordance to international ISO 15189 standard [22].

Four panels of European molecular quality controls (QCMD 2016 and 2017) were tested. RT assay results were correlated with the expected values. Only educational samples with values below 3 log_10_ IU/mL were not detected, thus slightly higher than LOD values we found, and found by other studies and those claimed by the manufacturer. In another work, Abbott RealTi*me* EBV run on a different system (*m*axCycle) detected all EBV positive samples in 2015 and 2016 QCMD panels including those below 3 log_10_ IU/mL [26].

The comparison of RT assay with the V1 assay we used in clinical practice, on clinical samples, showed similar results. Quantitative values of positive samples were highly correlated. Eight percent of samples were discrepant and corresponded to low EBV load values. Viral load values measured with the RT assay were on average 0.30 log_10_ IU/mL lower than those measured with the V1 assay suggesting that threshold values for therapeutic management might be adapted. Previously, comparison between the same two assays gave similar results on plasma samples collection [21]. Vinuesa et al. study also found a significant correlation between assays on 60 plasma specimens quantifiable by both assays (*r*^2^ = 0.669; *p* = < 0.0001) but a non-significant difference between the two assays (0.07 log UI/mL). A seemingly higher difference between the two assays in our series may reflect the higher complexity for extracting whole blood than plasma and may account also for the higher number of samples tested in our work.

The comparison of both assays in the follow-up of patients with EBV loads requiring rituximab treatment showed stackable curves. Results of the patients’ follow-up suggest also that the RT assay gave a better estimation of control of EBV reactivation as it remained positive longer than V1 assay after rituximab injection. However, in these patients, a change in rituximab treatment due to the use of RT assay instead of V1 assay seems unlikely. In our institution, a follow-up of the patients was made during several weeks after the routine implementation of the Abbott assay and a new therapeutic threshold was defined in agreement with the clinicians and based on our local experience. Higher sensitivity might be useful for EBV half-life determination with current treatments and future therapies and thus might help in defining treatment efficacy. Such a technique and others in the market using international standard should now be used for multicenter studies to define a clinical threshold for preemptive therapies that might help to optimize rituximab treatment, considering its potential side-effects.

## Conclusion

In conclusion, the RT assay is a reliable assay for EBV load in whole blood. Its sensitivity will enable to estimate the kinetics of EBV load and the impact of treatments to control EBV reactivations.

## Data Availability

The datasets analysed are available from the corresponding author on request.

## Abbreviations

CV: Coefficients of Variation
EBV: Epstein Barr Virus
HSCT: Hematopoietic Stem Cell Transplant
IS: International Standard
IU: International Unit
LLQ: Lower Limit of quantification
LOD: Limit of Detection
PTLD: Post Transplant Lymphoproliferative Disorders
QCMD: Quality Control for Molecular Diagnosis
RT assay: EBV RealTime Abbott assay
V1 assay: EBV PCR Kit V1 Abbott assay
WB: Whole Blood
